# Clinical characteristics and leptomeningeal collateral status in pediatric and adult patients with ischemic moyamoya disease

**DOI:** 10.1111/cns.13130

**Published:** 2019-04-13

**Authors:** Zhi‐Wen Liu, Cong Han, Hui Wang, Qian Zhang, Si‐Jie Li, Xiang‐yang Bao, Zheng‐Shan Zhang, Lian Duan

**Affiliations:** ^1^ Department of Neurosurgery, The Fifth Medical Center of Chinese PLA General Hospital The 307th Hospital of the Chinese People's Liberation Army Academy of Military Medical Science Beijing China; ^2^ Departments of Neurology and Neurosurgery, Xuanwu Hospital, Center of Stroke, Beijing Institute for Brain Disorders Capital Medical University Beijing China

**Keywords:** adult, leptomeningeal collateral, moyamoya disease, pediatric

## Abstract

**Aim:**

Previous studies have found significant differences in clinical characteristics between pediatric and adult moyamoya disease (MMD) patients, but few studies have focused on the factors underlying these differences. We aimed to investigate the differences in leptomeningeal collateral (LMC) status between pediatric and adult MMD patients and to analyze the effects of LMCs on clinical characteristics and therapeutic prognosis.

**Methods:**

We retrospectively analyzed 214 MMD patients from January 2014 to January 2016. Clinical characteristics and LMC status were compared between the pediatric and adult patients. LMC status was graded as good or poor depending on the retrograde flow from the posterior cerebral artery (PCA) on digital subtraction angiography (DSA).

**Results:**

A total of 83 pediatric and 131 adult (1:1.6) MMD patients were analyzed. Pediatric patients were more likely to experience a transient ischemic attack (81%), whereas adult patients were more likely to experience infarction (51%). Regarding the different MMD stages (the early, medium, and advanced stages corresponded to Suzuki stages 1‐2, 3‐4, and 5‐6, respectively), the prevalence of good LMC status was higher for pediatric patients than for adult patients in the early stage (*P* = 0.047) and the medium stage (*P* = 0.001), but there were no differences between these patient groups in the advanced stage (*P* = 0.547). Worse postoperative angiographic outcomes (*P* = 0.017) were found in adult patients than in pediatric patients in the medium stage. Poor LMC status had strong correlations with infarction (*P* < 0.001 and *P* = 0.017) and poor postoperative outcomes (*P* = 0.003 and *P* = 0.043) in both pediatric and adult patients.

**Conclusions:**

Pediatric MMD patients have greater patency and a greater ability to establish good LMC status than adult patients, and poor LMC status has a strong correlation with severe clinical symptoms and poor postoperative outcomes. LMC status may be an important factor in the differences in clinical characteristics and prognosis between pediatric and adult MMD patients.

## 1 INTRODUCTION

Moyamoya disease (MMD) is characterized by progressive stenosis or occlusion of the bilateral internal carotid arteries (ICAs) with unknown etiology, and numerous collateral vessels appear at the base of the brain.^1^ There is a bimodal peak age at onset for MMD, with peaks in childhood and adulthood, and the clinical features differ between these patients.^2^ In contrast to adult patients with MMD, who often present with cerebral infarction, pediatric patients with MMD typically exhibit transient ischemic attack (TIA).^3^ However, relatively little attention has been devoted to the causes of these differences.

Moyamoya disease has a chronic, irreversible, and progressive natural course. For MMD, progressive stenosis and occlusion of the principal intracranial artery lead not only to disease progression but also to the development of collateral circulation. Many studies have indicated that leptomeningeal collateral (LMC) status is a strong predictor of long‐term functional outcomes in stroke patients with large vessel intracranial occlusion^4^, ^5^, ^6^ and plays the most important role in the collateral supply of the ischemic cortex of the anterior cerebral artery (ACA) and middle cerebral artery (MCA) territory in MMD patients.^7^ Although aging is a major risk factor for poor LMC status,^8^ relatively little attention has been devoted to the differences and effects of LMC status in pediatric and adult MMD patients. Therefore, in this work, we sought to elucidate these differences and analyze their clinical value.

## 2 METHODS

The authors declare that all supporting data are available within the article and its online‐only Data Supplements.

### 2.1 Subjects

The ethics committee of our institution granted ethical approval for this retrospective study (ky‐2018‐6‐60) and waived written informed consent.

We retrospectively reviewed bilateral MMD patients who were treated in our department between January 2014 and January 2016. The patients included in this study met the following criteria: (a) all of the patients underwent digital subtraction angiography (DSA) and met the current diagnostic criteria recommended by the moyamoya disease guidelines from 2012^9^; (b) to exclude moyamoya syndrome, the patients were required to exhibit no history of systemic diseases, such as atherosclerosis or immune system disease^9^; (c) the patients had no history of prior bypass surgery; (d) patients with two or more atherogenic risk factors, such as hypertension, diabetes mellitus, dyslipidemia, heavy smoking, heavy drinking, and congenital anomaly, were excluded because of unclear influences on collateral circulation^10^; (e) patients with severe posterior cerebral artery (PCA) involvement (stage 3 and 4 according to the Mugikura staging system) were excluded because of disturbed blood supply to the LMCs^11^; (f) the patients’ symptoms at onset were transient ischemic attack (TIA) and infarction, and patients were excluded if the details of the first clinical event could not be confirmed or if their symptoms did not match the imaging findings; and (g) pediatric patients were defined as patients under 18 years old (≤18).

### 2.2 Clinical data collection

Demographic and clinical data were collected according to a standard protocol during the patient's first visit to our institution. The data included age, sex, onset symptoms, and age at onset of initial symptoms. Infarction was verified by magnetic resonance imaging (MRI) or computed tomography (CT). The progression of MMD was stratified into three stages with a modified Suzuki staging system.^1^ Suzuki stages 1‐2, 3‐4, and 5‐6 were recorded as the early, medium, and advanced stages of MMD, respectively.

### 2.3 Leptomeningeal collateral assessment

**Figure 1:**
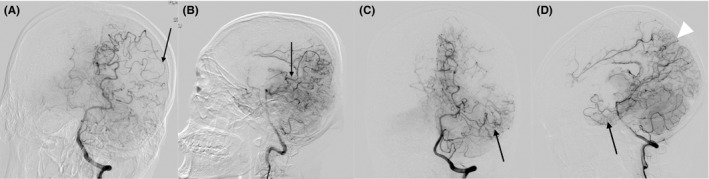
The leptomeningeal collaterals in patients with moyamoya disease

Because the lesions in MMD usually involve the bilateral cerebral hemispheres, many collateral grading methods for the healthy side do not apply to the side affected by MMD. In this study, LMC status was graded using DSA as follows: good LMC status was defined as anastomoses of the parieto‐occipital PCA branches to the ACA and MCA in the watershed zones and/or anastomoses of the anterior temporal PCA branches to the temporal MCA branches, and poor LMC status was defined as the absence of leptomeningeal anastomoses (Figures 1 A‐D). Angiographic findings and CT and MR images were evaluated by two radiologists who were blinded to the identities of the patients.

There were few DSA images of the cerebral arteries in normal patients. The LMC status in the normal hemispheres of 128 consecutive confirmed unilateral MMD patients (50 pediatric and 78 adult patients) who were treated in our department between January 2014 and January 2016 were used as a baseline. Good LMCs were found in only 2% (1/50) of normal hemispheres in pediatric patients and 1% (1/78) of normal hemispheres in adult patients. There was no difference in the LMC status in the normal hemisphere between the pediatric and adult patients (*P* = 0.742).

### 2.4 Treatment and follow‐up

Encephaloduroarteriosynangiosis (EDAS) was performed in 252 symptomatic hemispheres within 2 weeks after DSA, and the opposite hemisphere was usually revascularized 3 months after the first surgery, if required. Postoperative DSA was routinely performed 6‐9 months after surgery. The effect of revascularization through EDAS was graded according to the following system described by Matsushima et al^12^: Grade I, revascularization of less than 1/3 of the MCA distribution; Grade II, revascularization of between 1/3 and 2/3 of the MCA distribution; and Grade III, revascularization of over 2/3 of the MCA distribution.

Follow‐up was performed on a per‐hemisphere basis until the occurrence of a primary or secondary event (in that hemisphere) or the end of the study (November 2017). A standardized script was used to screen for interval stroke in phone interviews. The primary endpoint was the occurrence of infarction or bleeding in the symptomatic hemispheres. The secondary endpoint was death. Endpoint determination was performed by an independent experienced physician who was blinded to the DSA data and was based on a review of clinical and imaging data.

### 2.5 Statistical analyses

The Mann‐Whitney *U* test was used to compare continuous variables. The Pearson chi‐square test was used to compare categorical variables between groups. All analyses were performed with SPSS software, version 24.0 (International Business Machines Corp., Almond, NY, USA), and *P* < 0.05 was considered statistically significant.

## 3 RESULTS

### 3.1 Patient characteristics

A total of 279 consecutive confirmed bilateral MMD patients were identified in our hospital between January 2014 and January 2016. A total of 24 pediatric and 41 adult patients with severe posterior cerebral artery (PCA) involvement (stage 3 and 4 according to the Mugikura staging system) were excluded. In total, 214 MMD patients (pediatric: adult patients = 1:1.6) were finally included. The demographic features of the 83 pediatric patients (male: female = 1:1, 9.4 ± 3.5 years) and 131 adult patients (male: female = 1:1.6, 37.1 ± 9.2 years) are summarized in Table 1. The average interval from the onset of symptoms to angiography was 17.4 ± 19.1 months in pediatric patients and 23.7 ± 29.6 months in adult patients.

**Table 1:** Demographic data for the 214 bilateral MMD patients

Variables	Children (%)	Adults (%)	*P* value
Number of patients (pts)	83	131	
Age	9.4 ± 3.5	37.1 ± 9.2	
Sex			
Females	43 (52%)	81 (62%)	0.148
Males	40 (48%)	50 (38%)	
Clinical presentation (pts)			<0.001
TIA	67 (81%)	64 (49%)	
Infarction	16 (19%)	67 (51%)	
Suzuki stage (hps)			0.076
Early	31 (29%)	37 (24%)	
Medium	66 (62%)	90 (58%)	
Advanced	10 (9%)	29 (19%)	
Total	107	156	
LMC (hps)			
Good	92 (86%)	106 (68%)	0.001
Poor	15 (14%)	50 (32%)	
Matsushima Grade (hps)			0.002
1	36 (35%)	74 (50%)	
2	27 (26%)	43 (29%)	
3	41 (39%)	31 (21%)	
Total	104	148	
Postoperative stroke (hps)	5	8	0.833

hps, hemispheres; pts, patients.

### 3.2 Difference in clinical characteristics between pediatric and adult patients with ischemic MMD

The clinical presentations were significantly different between pediatric and adult patients (*P* < 0.001). Pediatric patients were more likely to have TIA (67/83, 81%), while adult patients were more likely to have infarction (67/131, 51%). A total of 263 (107 in pediatric patients and 156 in adult patients) symptomatic hemispheres were analyzed. The Suzuki stages showed no significant difference between adult and pediatric patients with MMD (*P* = 0.076). In the early and medium stages, pediatric patients were more likely to present with TIA (both *P* < 0.001) than adult patients, but there was no significant difference in clinical symptoms between these patient groups in the advanced stage (*P* = 0.651).

Encephaloduroarteriosynangiosis was performed in 252 symptomatic hemispheres in 246 patients (104 hemispheres in 81 pediatric patients and 148 hemispheres in 125 adult patients). Worse postoperative angiographic outcomes (*P* = 0.017) were found in adult patients than in pediatric patients in the medium stage.

During an average follow‐up time of 27.57 ± 9 months, five infarctions occurred in pediatric patients, and seven infarctions and one bleeding event occurred in adult patients, but no patients died. There was no significant difference in the postoperative stroke rate between pediatric and adult patients with MMD (*P* = 0.833).

### 3.3 Difference in leptomeningeal collateral status between pediatric and adult patients with ischemic MMD

**Figure 2:**
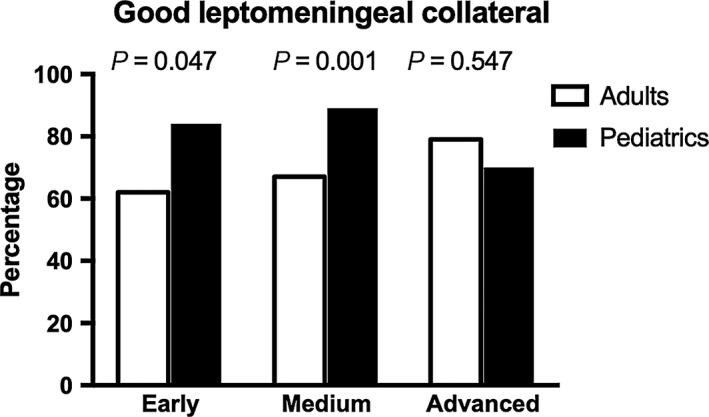
The prevalence of good leptomeningeal collaterals in pediatric and adult patients with ischemic moyamoya disease in different Suzuki stages

A significant difference in LMC status was found between the two patient groups; the prevalence of good LMC status in pediatric patients with MMD was higher than that in adult patients (*P* = 0.002, Table 1). The LMC status in different stages was further compared in Table 2. There was no significant difference in LMC status in the advanced stage (Suzuki stage 5‐6) between the two patient groups (*P* = 0.547), but in the early (Suzuki stage 1‐2) and medium (Suzuki stage 3‐4) stages, pediatric patients were more likely to have good LMCs (*P* = 0.047 and *P* = 0.001; Figure 2) than adult patients.

**Table 2:** Differences in clinical presentation and LMC status between children and adults in different Suzuki stages

	Early stage			Medium stage			Advanced stage		
	Children	Adults	*P*	Children	Adults	*P*	Children	Adults	*P*
Clinical presentation (hps)			<0.001			<0.001			0.651
TIA	29 (94%)	21 (57%)		56 (85%)	48 (53%)		6 (60%)	15 (52%)	
Infarction	2 (6%)	16 (43%)		10 (15%)	42 (47%)		4 (40%)	14 (48%)	
Total	31	37		66	90		10	29	
LMC (hps)			0.047			0.001			0.547
Good	26 (84%)	23 (62%)		59 (89%)	60 (67%)		7 (70%)	23 (79%)	
Poor	5 (16%)	14 (38%)		7 (11%)	30 (33%)		3 (30%)	6 (21%)	
Matsushima Grade (hps)									
1	12 (40%)	18 (55%)	0.061	20 (31%)	43 (49%)	0.017	4 (40%)	13 (46%)	0.503
2	6 (20%)	11 (33%)		19 (30%)	23 (76%)		2 (20%)	9 (32%)	
3	12 (40%)	4 (12%)		25 (39%)	21 (24%)		4 (40%)	6 (21%)	
	30	33		64	87		10	28	

### 3.4 Association between leptomeningeal collateral status and clinical characteristics

The associations of LMC status with clinical symptoms and prognosis in pediatric and adult patients with MMD are compared in Table 3. Good LMC status was significantly correlated with TIA in pediatric and adult patients (*P* < 0.001 and *P* = 0.017).

**Table 3:** Associations between clinical characteristics and leptomeningeal collateral status in pediatrics and adults with moyamoya disease

	Pediatrics with ischemic MMD			Adults with ischemic MMD		
	Good LMC	Poor LMC	*P*	Good LMC	Poor LMC	*P*
Clinical presentation (hps)			<0.001			0.017
TIA	85 (92%)	7 (44%)		64 (60%)	20 (40%)	
Infarction	7 (8%)	9 (56%)		42 (40%)	30 (60%)	
Total	92	16		106	50	
Matsushima Grade (hps)			0.305			0.133
1	32 (36%)	4 (29%)		53 (51%)	21 (47%)	
2	24 (27%)	3 (21%)		35 (34%)	8 (18%)	
3	34 (38%)	7 (50%)		15 (15%)	16 (36%)	
Total	90	14		103	45	
Postoperative stroke (hps)	2	3	0.003	3	5	0.043

hps, hemispheres; LMC, leptomeningeal collateral; MMD, moyamoya disease.

The postoperative angiographic findings showed that there was no correlation between Matsushima stage and LMC status in pediatric (*P* = 0.285) or adult patients (*P* = 0.133, Table 2) with MMD. However, poorer LMC status was associated with significantly higher postoperative stroke rates in pediatric (*P* = 0.003) and adult patients (*P* = 0.043) with MMD.

## 4 DISCUSSION

In this study, we found that there were significant differences in clinical symptoms and LMC status between pediatric and adult MMD patients and that LMC status had a strong correlation with the severity of clinical symptoms and postoperative prognosis. In addition, in the early and medium stages of MMD, pediatric patients were more likely to have good LMCs than adult patients. This finding suggested that pediatric MMD patients might have the ability to establish intracranial collateral circulation more easily than adult patients in the early and medium stages and that the collateral circulation status plays an important role in the clinical characteristics and prognosis of MMD patients.

There is a bimodal age distribution of moyamoya disease, with the first peak occurring in the pediatric population, typically in the first decade of life, and a second peak occurring between 30 and 40 years of age.^13^ Pediatric patients primarily suffer from ischemia symptoms, and adult patients typically experience intracranial hemorrhage.^2^ A Korean study in 2010 revealed that ischemic symptoms, especially TIAs, are predominant (70%)^14^ in pediatric patients. Another epidemiological study in Japan (2012) showed that although TIA and infarction accounted for 60% of all cases, TIA mainly occurred in pediatric patients, while infarction had a higher incidence in adult patients.^15^ In our study, we found that TIA was the major symptom in pediatric patients, whereas infarction was more prevalent among adult patients. Our results are consistent with those of previous studies and indicate that differences in clinical symptoms exist between pediatric and adult patients. The causes of these differences remain unclear, and few studies have investigated this issue.

The development of abundant collateral circulation is more common in MMD, a chronic progressive cerebrovascular disease, than in other ischemic cerebrovascular diseases. Previous studies have found that the collateral circulation, especially from the leptomeningeal system, plays the most important role in the collateral supply for MMD patients.^7^ The presence of collateral flow via the posterior communicating artery is associated with a low prevalence of border zone infarcts,^16^ while stenosis or occlusion of the PCA is often associated with a high stroke rate in MMD patients.^17^ Almost no LMCs from the PCA were observed in normal hemispheres without ACA or MCA stenosis/occlusion. In our study, we found that the prevalence of good LMCs was higher in pediatric patients than in adult patients during the early and medium MMD stages, and poor LMC status had a strong correlation with severe clinical symptoms, such as infarction, in the medium stage. This finding reveals that the intracranial collateral circulation plays an important role in the differences in clinical symptoms between pediatric and adult MMD patients. Therefore, we conclude that good intracranial collateral compensation could improve cerebrovascular reserve and tolerance to cerebral ischemia and lead to milder symptoms at onset and lower incidence rates of stroke in pediatric patients.

The mechanism for the differences in LMC status between pediatric and adult MMD patients in the same stage remains unclear. Experimental studies have shown that aging causes a significant decrease in LMCs, as well as increased tortuosity and vascular resistance in leptomeningeal vessels.^18^, ^19^ It is also well established from human studies that age has a detrimental effect on cerebrovascular reactivity.^20^ Therefore, age may be an important influencing factor for collateral development. Second, focal cerebral ischemia may stimulate collateral growth via the secretion of cytokines, such as angiogenic peptides^21^ and vascular endothelial growth factor (VEGF).^22^ Some studies have shown that VEGF levels are lower in aged individuals than in young individuals.^23^ Third, common concomitant diseases in adult MMD patients, such as hypertension, have been shown to affect the development of collaterals in rats.^24^

In this study, we also discovered that poor LMC status had a strong correlation with postoperative stroke. Previous studies have shown that LMCs are important for hemodynamic status.^25^ Therefore, before postoperative collaterals were established by EDAS, LMCs may have played the most important role in the collateral supply. How the LMC status may be improved, especially in the early and medium stages, might be an important future research field and a new therapeutic target for improving clinical symptoms and prognosis.

There are several limitations of our work. One limitation is that all patients were enrolled from a single neurosurgery center, and potential selection bias related to regions and race may have occurred. Second, we did not consider the impact of other collateral pathways, such as transdural collaterals. However, because the LMCs from the PCAs to the anterior circulation are the earliest patent and most common collaterals^26^ and due to the barriers of the cerebrospinal fluid and skull, it was difficult to establish collaterals between the external carotid artery (ECA) and the ICA.^27^ Therefore, we believe that LMCs might be more important in the early and medium MMD stages in both pediatric and adult patients. In our study, we described only the association between LMCs and clinical characteristics. Further studies are needed to explore this relationship, such as investigations of cerebral hemodynamics in moyamoya disease using multiple inversion time arterial spin labeling MRI. Moreover, the mechanisms underlying the difference in the prevalence of good LMCs between pediatric and adult patients remain unclear and require further study.

## 5 CONCLUSIONS

Pediatric MMD patients are more likely than adult patients to exhibit good LMC status in the early and medium stages, and poor LMC status has a strong correlation with severe clinical symptoms and poor postoperative outcomes. Assessments of systematic collateral circulation may help to better evaluate disease progression and prognosis in MMD patients. Further research on the mechanisms of collateral patency may help us understand how to improve collateral status and provide a novel potential therapeutic approach for MMD.

## CONFLICTS OF INTEREST

The authors declare that there are no conflicts of interest.
